# Accidentally Extruded Calcium Hydroxide Into a Cystic Lesion Associated With an Adjacent Tooth—A Case Report

**DOI:** 10.1002/ccr3.70762

**Published:** 2025-09-28

**Authors:** Emmanuel Mazinis, Nikolaos Tsanidis, Vasilios Thomaidis

**Affiliations:** ^1^ Dept of Endodontology, Dental School Aristotle University of Thessaloniki University Campus Thessaloniki Greece; ^2^ Private Practice Alexandroupolis Greece; ^3^ Dept of Anatomy, Medical School Democritus University of Thrace Alexandroupolis Greece

**Keywords:** calcium hydroxide, cyst, endodontic treatment, extrusion barium sulfate

## Abstract

A rare case of pulp necrosis and apical resorption of a second premolar, caused by a cystic lesion from a first molar, led to calcium hydroxide extrusion. CBCT imaging, combined with clinical and historical findings, enabled accurate assessment of the lesion, resorption extent, and extruded material to guide treatment.

## Graphical abstract

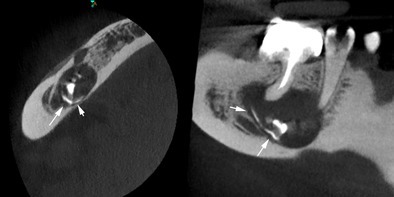

## 1 Introduction

During endodontic treatment, it is essential to ensure that interappointment intracanal medicaments and filling materials remain confined within the root canal system. The extrusion of filling materials, particularly in the maxillary and mandibular posterior teeth, poses a significant iatrogenic risk to adjacent anatomical structures, such as the maxillary sinus or the inferior alveolar nerve [1].

Although endodontic materials are generally biocompatible, their presence beyond the apex can act as a foreign body, leading to irritation of the periapical tissues. This irritation may result in treatment failure, chronic inflammation (Nair, 2003; [2]), delayed tissue repair, or various postoperative complications [3]. The radiopacity of materials used in endodontics facilitates the detection of extrusion and the assessment of its extent.

Radiographic evaluation plays a crucial role in diagnosing dental and periapical conditions. Two‐dimensional imaging, such as intraoral and panoramic radiographs, often lacks the precision required to determine the exact position of foreign materials relative to the surrounding space and adjacent structures [4]. Cone‐beam computed tomography (CBCT) clearly provides the advantage of more accurate localization of the position, size (Hassan, 2009; [5]), and shape of materials detected in the periapical tissues. CBCT imaging can sometimes display radiopaque artifacts caused by prior procedures, so a careful evaluation of the imaging data together with the clinical findings is necessary for an accurate diagnosis [6].

In the presented case report, pulp necrosis of an intact tooth occurred as a result of a cystic lesion originating from an adjacent tooth. This condition led to apical root resorption and loss of the apical constriction, allowing the calcium hydroxide placed as an intracanal medicament to extrude into the periapical tissues.

## 2 Case History

**FIGURE 1:**
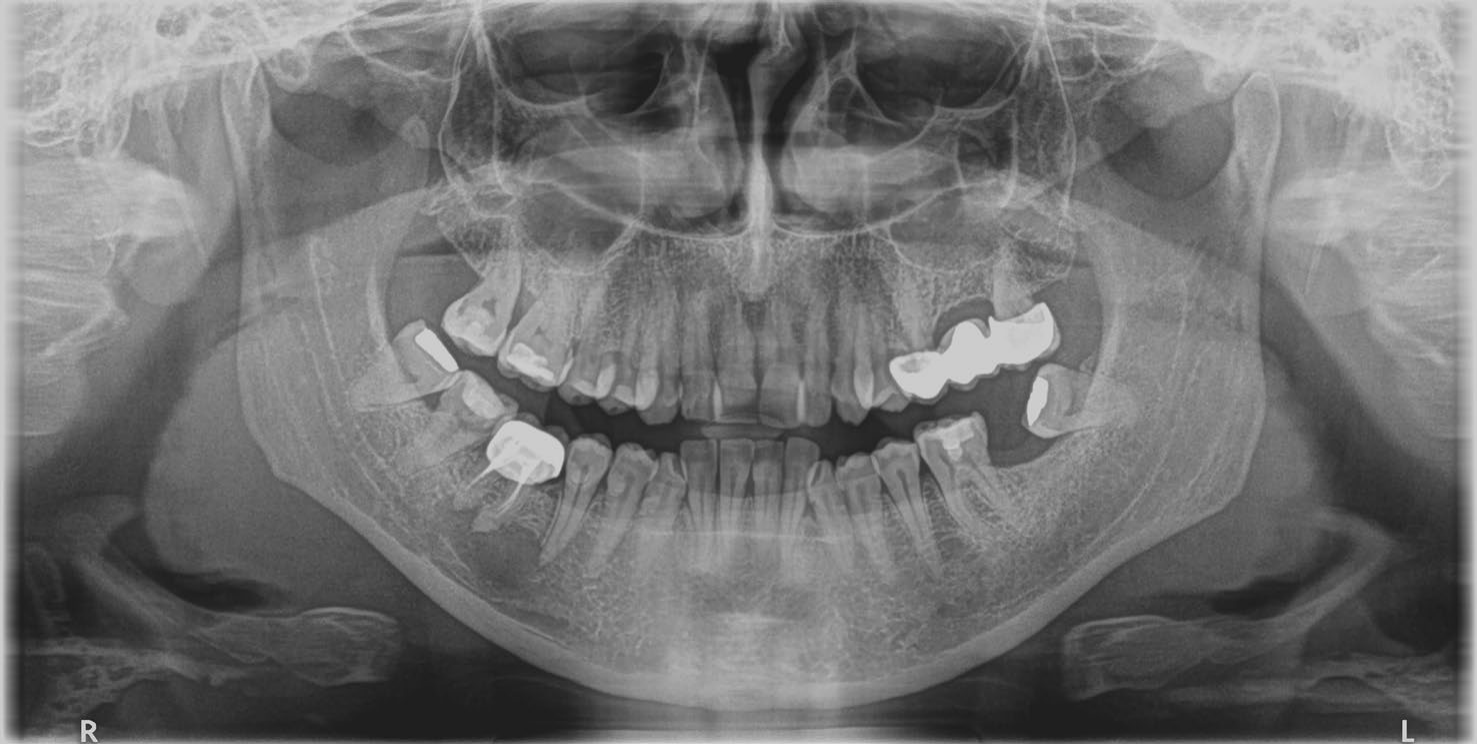
Panoramic radiograph obtained 2 years before the clinical examination.

A healthy 46‐year‐old male presented with pain on percussion in the region of the right lower second premolar (#45). The patient had undergone a panoramic radiograph 2 years ago, which revealed an incomplete root canal treatment of tooth #46 and a large cystic lesion extending into the surrounding tissues, including the apex of the second premolar (Figure 1).

**FIGURE 2:**
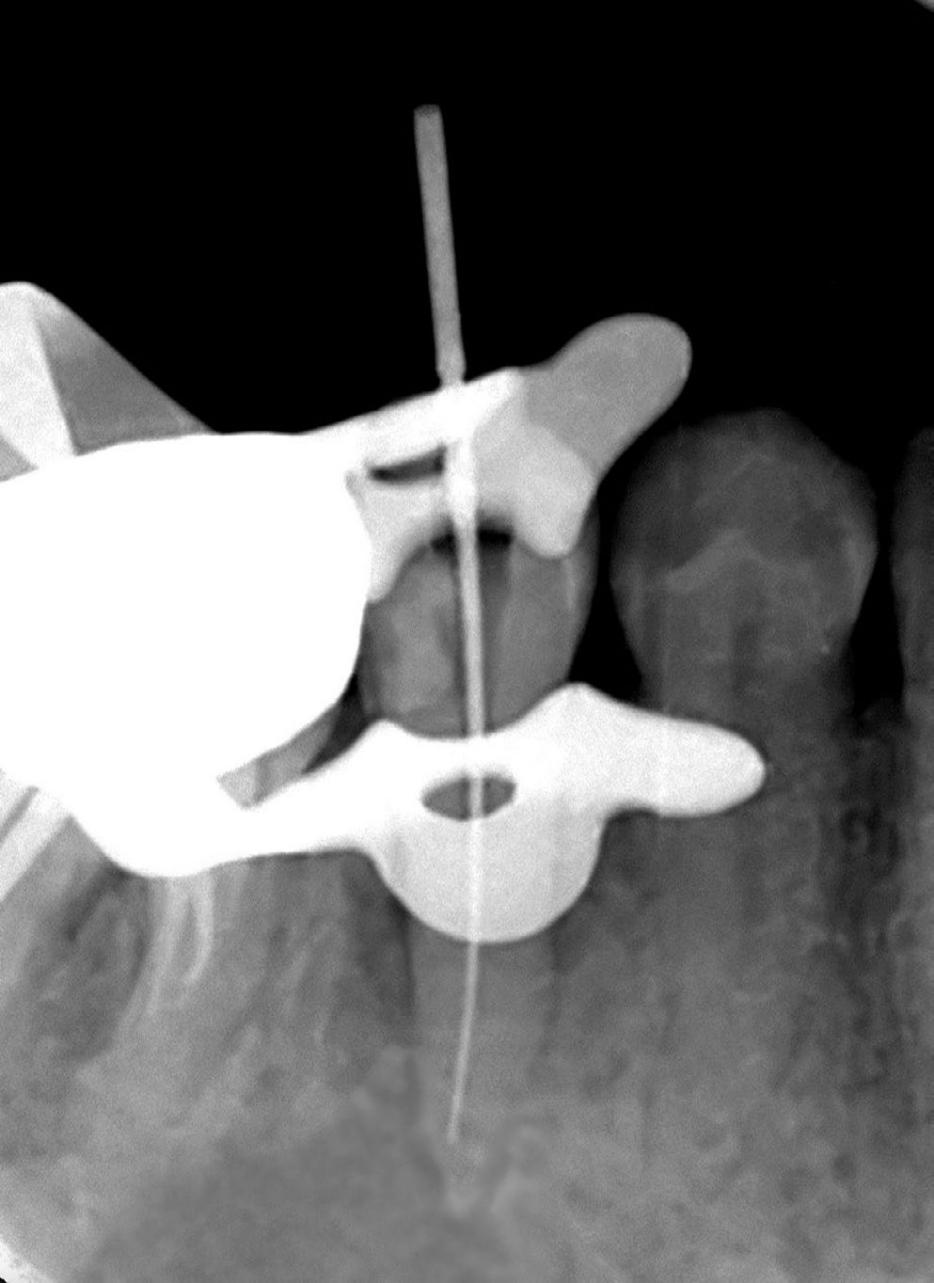
Determination of root canal length. The premolar shows evidence of resorption on the distal aspect of the root apex.

On clinical examination, the second premolar (#45) exhibited significant sensitivity to percussion, with no clinical evidence of carious lesions or history of trauma. The tooth was negative to pulp sensitivity tests. Once the pulp chamber was located, an orifice shaper was used to widen the access cavity, and canal patency was confirmed using a #10 K‐file. Root length was calculated using an apex locator and root length determination radiograph (Figure 2). Motor‐driven instruments with a taper of 0.4 in Sizes 15–45 were used for canal instrumentation.

The root canal was irrigated with 5 mL of 3% sodium hypochlorite and 5 mL of 17% EDTA between file changes. Calcium hydroxide containing barium sulfate was used as an interappointment medicament (Calacept, Directa) with a K‐file #25 without applying pressure. A follow‐up appointment was scheduled to complete the endodontic treatment, but the patient returned the next day with severe pain and swelling and significant tenderness to percussion in both molars and premolars. The patient also reported mild numbness of the lip. The temporary filling of the premolar was removed for immediate decompression, which resulted in drainage of a large amount of seropurulent exudate. The patient was prescribed antibiotic therapy and asked to undergo a new panoramic radiograph to determine if the cystic lesion had increased in size compared to its extent on the previous radiograph taken 2 years earlier.

**FIGURE 3:**
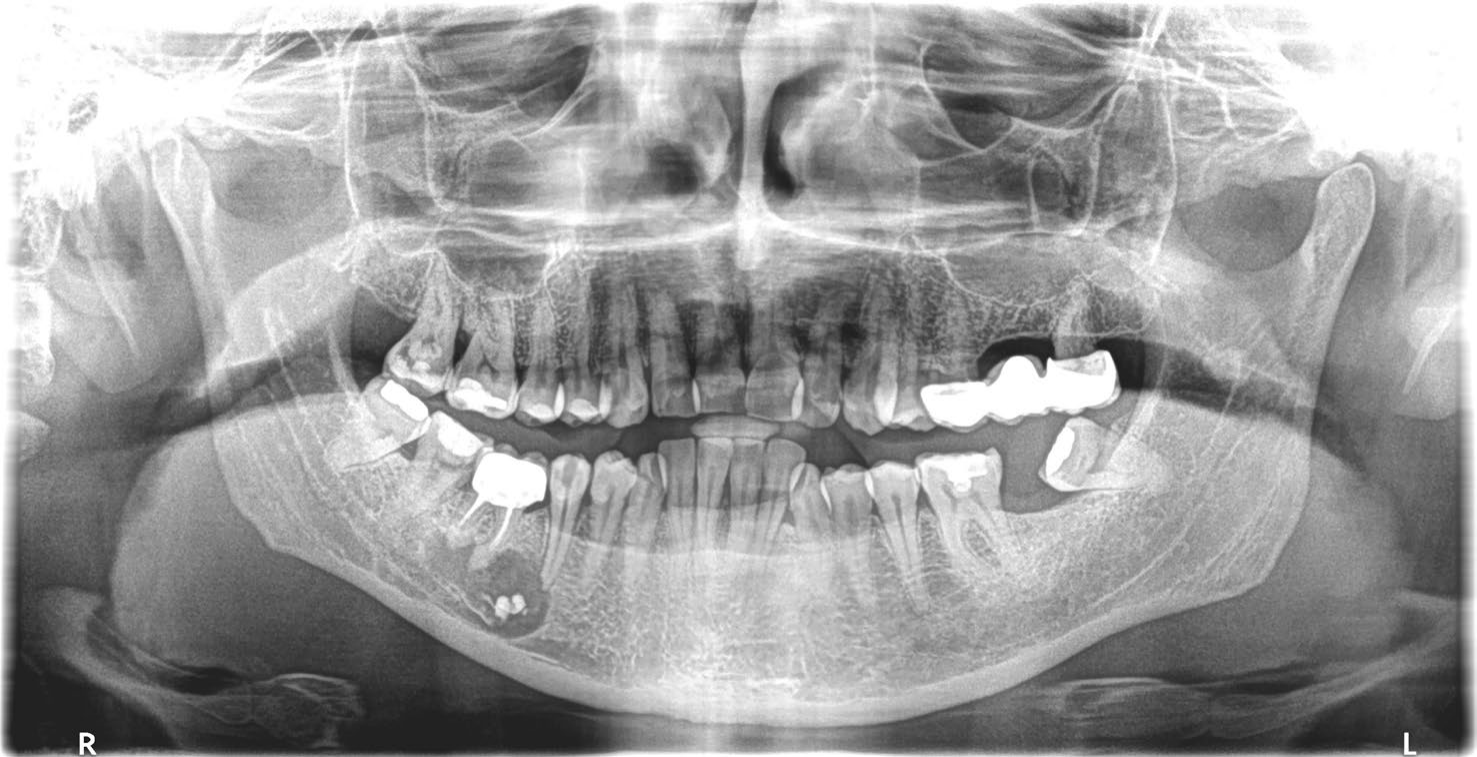
Panoramic radiogrpaph after decompression of tooth #45. The radiograph reveals the extrusion of calcium hydroxide into the cystic lesion.

**FIGURE 4:**
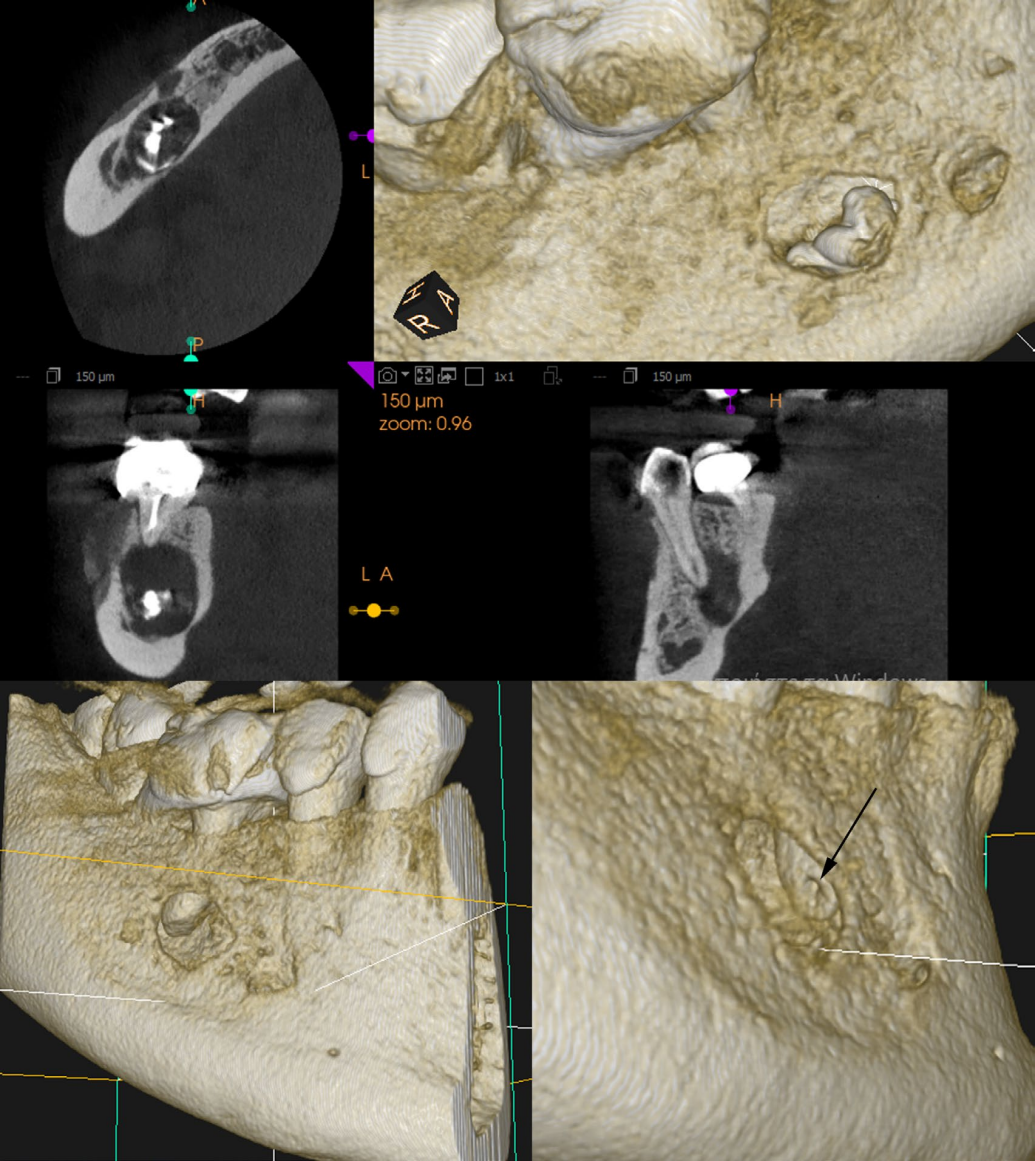
CBCT three‐dimensional reconstruction of the lesion and radiopaque materials. Apex resorption and widening of the apical constriction are evident (arrow).

**FIGURE 5:**
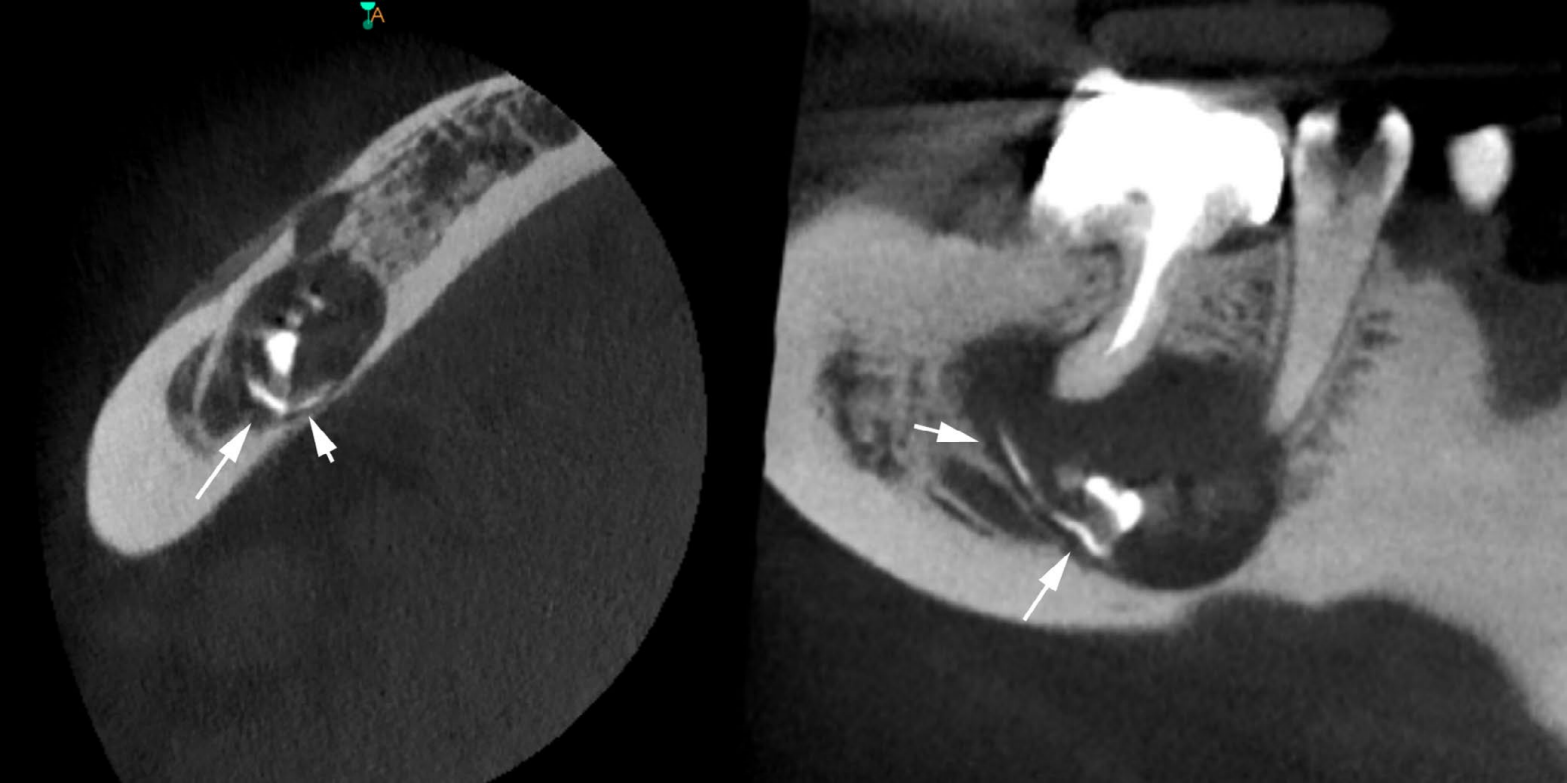
Radiopaque lines in the inner borders of the lesion (arrows).

The second panoramic radiograph (Figure 3) revealed extensive extrusion of calcium hydroxide into the cystic lesion in close proximity to the inferior alveolar nerve and mental foramen. Given these findings, a CBCT scan was requested to determine the extent of material extrusion and its relationship to anatomical landmarks. The CBCT provided a detailed view of the cystic lesion and calcium hydroxide extrusion. The scan showed three small radiopaque calcium hydroxide deposits within the cystic lesion. In addition, a radiopaque outline of calcium hydroxide was observed at the periphery of the lesion, extending to its internal margins (Figures 4, 5). The distribution of calcium hydroxide at the margins of the lesion was not visible on the panoramic radiograph. The cyst extended into the jaw bone in proximity to the mental foramen without evidence of the inferior alveolar nerve. Furthermore, resorption of the apical area of the second premolar was also evident, suggesting that a resorptive process caused its necrosis and loss of apical restriction was the reason for calcium hydroxide extrusion (Figure 4, arrow).

## 3 Treatment

The treatment plan included the obturation of the second premolar, followed by the surgical removal of the cyst, the foreign bodies, and tooth #46. Lip numbness was resolved the following day after decompression. The root canal was re‐instrumented, and an apical stop was created by instrumentation with K‐files up to # 60, 1.5 mm lower than the original length determination in order to avoid overfilling [7]. Hand files were used in the apical area to provide improved tactile feedback and greater control during instrumentation.

**FIGURE 6:**
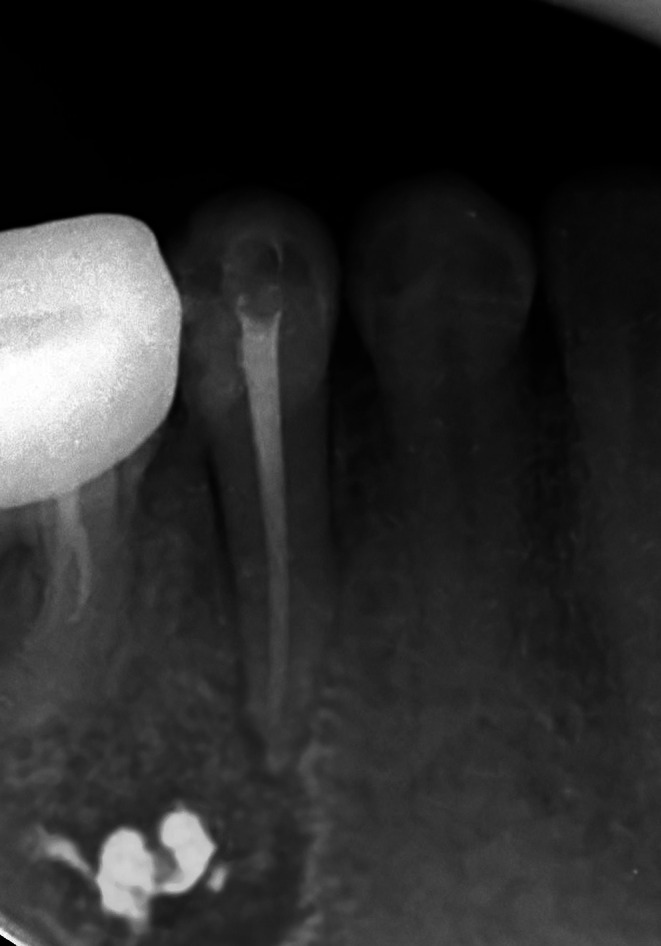
Final radiograph of the secod premolar before surgical intervention.

One week later, the second premolar was asymptomatic and was obturated with #55 guttapercha points and AH‐26 sealer (Dentsply Maillefer, Switzerland), using the lateral condensation technique to avoid material extrusion (Figure 6). The access cavity was temporarily restored with a cotton pellet, and a final restoration with composite resin was planned.

**FIGURE 7:**
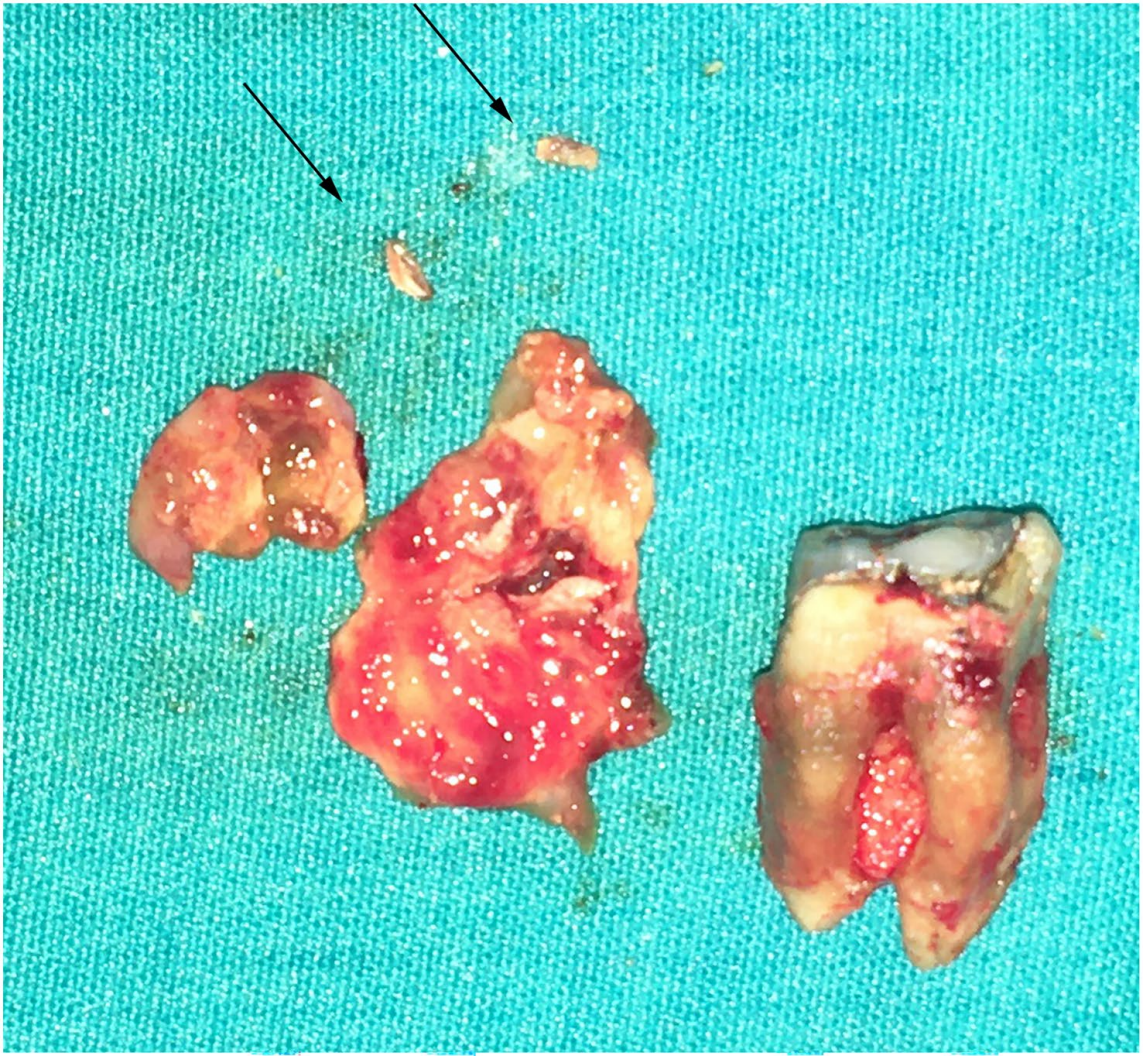
The extracted tooth #46, the cystic lesion and the remnants of calcium hydroxide (arrows).

**FIGURE 8:**
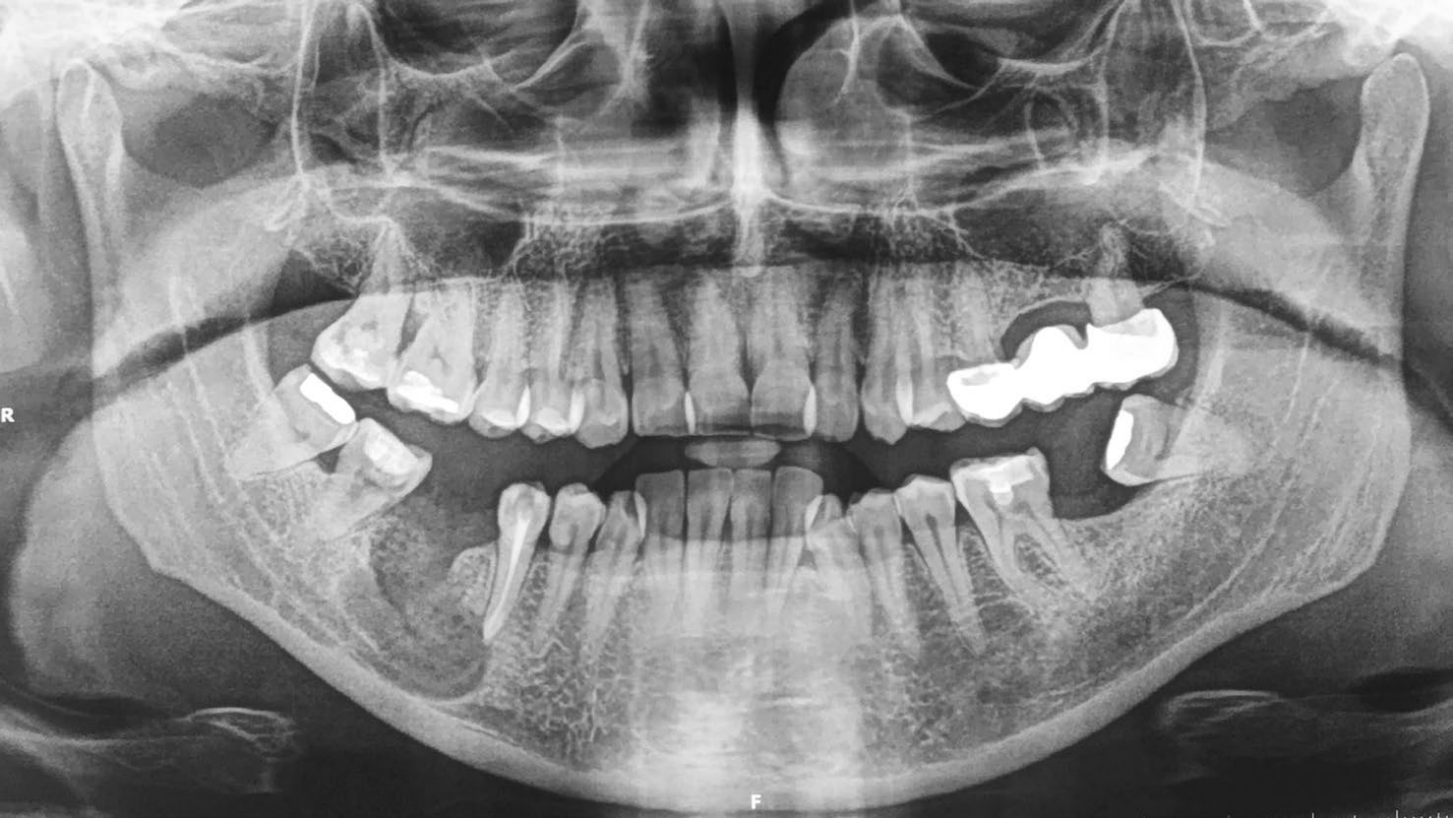
Panoramic radiograph 2 days after surgery. All radiopaque materials were removed.

During surgery, tooth #46 was extracted, and the cystic lesion was surgically accessed with particular care to avoid injury to the inferior alveolar and mental nerves (Figure 7). The cystic remnants were preserved and sent for histopathological examination. Calcium hydroxide remnants were located and removed as small masses of coagulated brittle material located within the lesion (Figure 7, arrows). A panoramic radiograph taken 2 days later confirmed the complete removal of all radiopaque materials (Figure 8).

Histopathological sections showed a cystic lesion lined by hyperplastic stratified squamous epithelium. The fibrous connective tissue wall showed chronic inflammatory infiltrates, predominantly lymphocytes, plasma cells, and multinucleated giant cells. There was no evidence of malignancy. The calcium hydroxide remnants were brittle in consistency.

**FIGURE 9:**
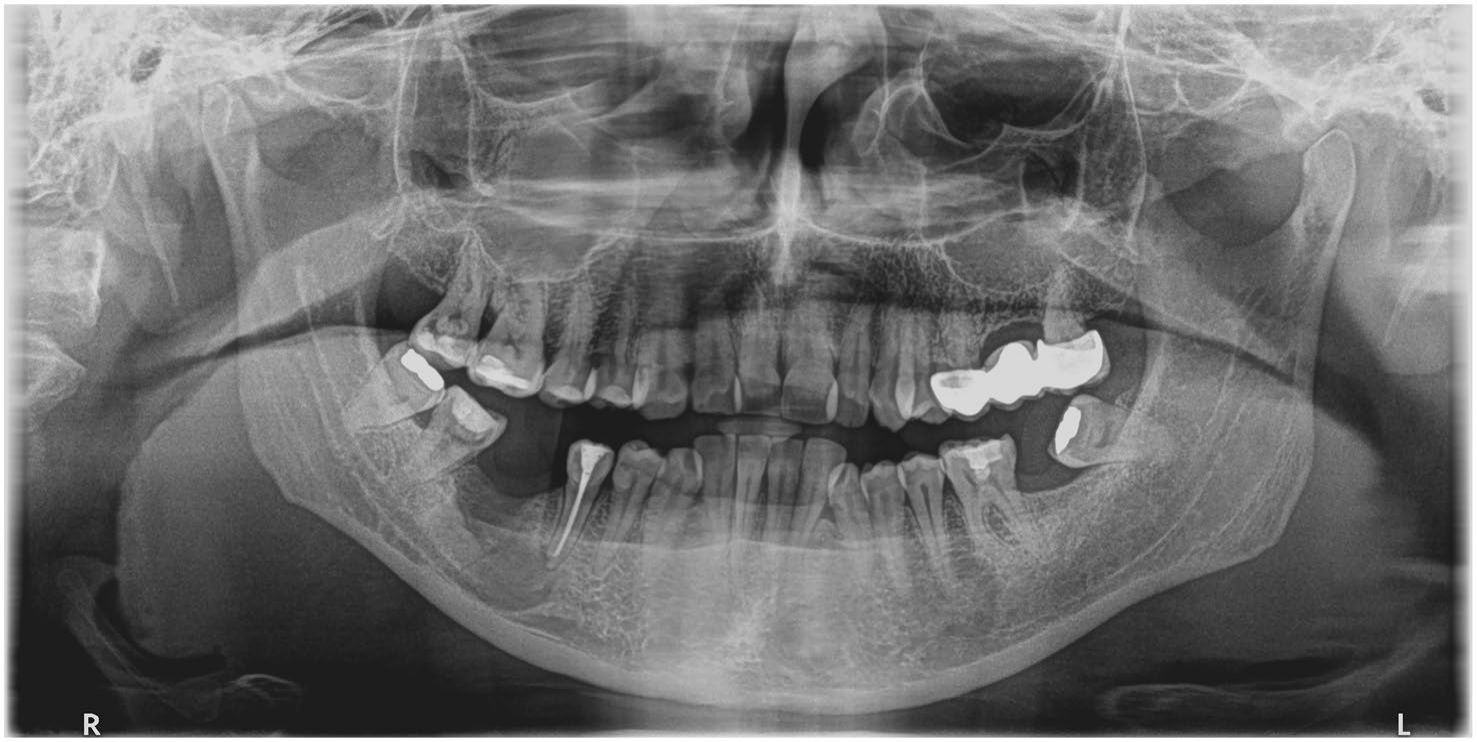
Three months recall panoramic radiograph.

At the 3‐month follow‐up, the patient remained asymptomatic, with evidence of healing of the lesion by formation of osseous tissue both around the premolar and the surgical area (Figure 9).

## 4 Discussion

Calcium hydroxide is a widely used intracanal medication due to its high pH, which provides antimicrobial effects and promotes healing. Intentional extrusion of calcium hydroxide into cystic lesions has been reported to promote lesion resolution or resorption, although the results of such approaches remain uncertain (Ioannidis, 2010). Most commercial calcium hydroxide formulations contain radiopaque excipients, such as barium sulfate or iodoform, which make them visible on radiographs. In this case, the radiopaque excipient of the formulation used was barium sulfate. The resorption time of calcium hydroxide in tissues can vary from a few weeks to several months [8]. In many cases, even after the calcium hydroxide has been resorbed, only the radiopaque excipients remain visible for an extended period [9].

Calcium hydroxide extrusion can lead to serious complications and adverse tissue reactions and tissue damage, ranging from local inflammatory reactions to more severe complications such as persistent pain, swelling, severe foreign body reactions, and even nerve damage or paresthesia [3, 10, 11, 12]. Extrusion can occur particularly when excessive pressure is applied during intracanal medicament placement or is facilitated by the morphology of the tooth and its apical anatomy [13].

Pulp necrosis of an intact adjacent tooth can occur as a result of a periapical lesion in an adjacent tooth. The mechanism of necrosis in adjacent teeth has been attributed to either pressure or bacterial retrograde entry from the lesion through the apical foramen or lateral canals [14, 15, 16], although cases of necrosis and apical resorption and extrusion of calcium hydroxide have not been reported.

In the current literature, there are no reported cases where the extrusion of calcium hydroxide was caused by apical root resorption resulting from a cystic lesion originating from an adjacent tooth. In most cases, extrusion occurs either accidentally due to excessive pressure during placement or intentionally as part of treatment for extensive lesions (Byun 2014; [2, 9, 17]).

The use of CBCT may facilitate the diagnosis and identification of resorptive lesions that are not readily apparent on two‐dimensional imaging. CBCT is the imaging modality of choice in cases involving extrusion of radiopaque materials into the periapical tissues. In cases of necrosis of an adjacent tooth without clinical signs of pathology, the use of CBCT is considered diagnostically essential to assess the extent of the lesion and to investigate the possibility of a non‐dentigerous etiology [18].

The clinical history of the use of calcium hydroxide in Tooth 45 as an intracanal medicament aimed to identify the type of radiopaque material that was extruded. Differentiation of the radiopaque materials in the periradicular tissues is not possible, as the radiographic appearance of extruded root canal sealer, metal fragments, or even barium sulfate residues is similar. The potential for radiopaque artifacts on radiographs, particularly in the presence of extruded endodontic materials, necessitates a multimodal diagnostic approach. The contribution of CBCT was significant, providing details of the proximity of the lesion to nerve structures, the position and distribution of the calcium hydroxide, and the radiographic appearance of the premolar apex.

## Author Contributions

**Emmanuel Mazinis:** conceptualization, data curation, formal analysis, investigation, methodology, project administration, resources, software, supervision, validation, visualization, writing – original draft, writing – review and editing. **Nikolaos Tsanidis:** conceptualization, data curation, formal analysis, investigation, methodology, project administration, resources, software, supervision, validation, visualization, writing – original draft, writing – review and editing. **Vasilios Thomaidis:** conceptualization, data curation, formal analysis, investigation, methodology, project administration, resources, software, supervision, validation, visualization, writing – original draft, writing – review and editing.

## Consent

Written informed consent was obtained from the patient to publish this report in accordance with the journal's patient consent policy.

## Conflicts of Interest

The authors declare no conflicts of interest.

## Data Availability

Data available on request from the authors.
